# Circulating tumour DNA in metastatic breast cancer to guide clinical trial enrolment and precision oncology: A cohort study

**DOI:** 10.1371/journal.pmed.1003363

**Published:** 2020-10-01

**Authors:** Andjelija Zivanovic Bujak, Chen-Fang Weng, Maria João Silva, Miriam Yeung, Louisa Lo, Sarah Ftouni, Cassandra Litchfield, Yi-An Ko, Keilly Kuykhoven, Courtney Van Geelen, Sushma Chandrashekar, Mark A. Dawson, Sherene Loi, Stephen Q. Wong, Sarah-Jane Dawson

**Affiliations:** 1 Peter MacCallum Cancer Centre, Melbourne, Victoria, Australia; 2 Sir Peter MacCallum Department of Oncology, The University of Melbourne, Melbourne, Victoria, Australia; 3 Centre for Cancer Research, The University of Melbourne, Melbourne, Victoria, Australia; The Francis Crick Institute, UNITED KINGDOM

## Abstract

**Background:**

Metastatic breast cancer (mBC) is a heterogenous disease with increasing availability of targeted therapies as well as emerging genomic markers of therapeutic resistance, necessitating timely and accurate molecular characterization of disease. As a minimally invasive test, analysis of circulating tumour DNA (ctDNA) is well positioned for real-time genomic profiling to guide treatment decisions. Here, we report the results of a prospective testing program established to assess the feasibility of ctDNA analysis to guide clinical management of mBC patients.

**Methods and findings:**

Two hundred thirty-four mBC patients (median age 54 years) were enrolled between June 2015 and October 2018 at the Peter MacCallum Cancer Centre, Melbourne, Australia. Median follow-up was 15 months (range 1–46). All patient samples at the time of enrolment were analysed in real time for the presence of somatic mutations. Longitudinal plasma testing during the course of patient management was also undertaken in a subset of patients (*n* = 67, 28.6%), according to clinician preference, for repeated molecular profiling or disease monitoring. Detection of somatic mutations from patient plasma was performed using a multiplexed droplet digital PCR (ddPCR) approach to identify hotspot mutations in *PIK3CA*, *ESR1*, *ERBB2*, and *AKT1*. In parallel, subsets of samples were also analysed via next-generation sequencing (targeted panel sequencing and low-coverage whole-genome sequencing [LC-WGS]). The sensitivity of ddPCR and targeted panel sequencing to identify actionable mutations was compared. Results were discussed at a multidisciplinary breast cancer meeting prior to treatment decisions. ddPCR and targeted panel sequencing identified at least 1 actionable mutation at baseline in 80/234 (34.2%) and 62/159 (39.0%) of patients tested, respectively. Combined, both methods detected an actionable alteration in 104/234 patients (44.4%) through baseline or serial ctDNA testing. LC-WGS was performed on 27 patients from the cohort, uncovering several recurrently amplified regions including 11q13.3 encompassing *CCND1*. Increasing ctDNA levels were associated with inferior overall survival, whether assessed by ddPCR, targeted sequencing, or LC-WGS. Overall, the ctDNA results changed clinical management in 40 patients including the direct recruitment of 20 patients to clinical trials. Limitations of the study were that it was conducted at a single site and that 31.3% of participants were lost to follow-up.

**Conclusion:**

In this study, we found prospective ctDNA testing to be a practical and feasible approach that can guide clinical trial enrolment and patient management in mBC.

## Author summary

**Why was this study done?:**

- Comprehensive real-time genomic profiling is becoming increasingly important to guide therapeutic decisions in metastatic breast cancer (mBC).
- Tumour biopsies, often taken at the time of primary disease diagnosis, remain the main source of tumour genomic material but may not reliably capture the genomic heterogeneity of a patient’s tumour in space and/or time.
- Circulating tumour DNA (ctDNA) offers a minimally invasive alternative to complement traditional tumour biopsies for molecular profiling.

**What did the researchers do and find?:**

- We established a prospective ctDNA testing program for patients with mBC to assess the feasibility of this approach to guide patient management.
- Using a combination of different genomic approaches, actionable alterations were identified in 44% of patients, and clinical management was directly altered by ctDNA testing in 39% of these cases.

**What do these findings mean?:**

- Our experience of implementing a prospective ctDNA testing program in mBC demonstrates the feasibility and value of this approach to direct patient management and supports the routine incorporation of ctDNA for molecular profiling in this disease.

## Introduction

Breast cancer is the most common cancer and the leading cause of cancer-related death in women worldwide [1]. Although targeted therapies for estrogen receptor–positive (ER+) and *ERBB2*-amplified (human epidermal growth factor receptor 2–positive [HER2+]) breast cancers have become the mainstay of treatment over several decades, a rapidly growing number of novel agents are now emerging whose effectiveness may depend on specific genomic aberrations. Examples include activating mutations in *PIK3CA*, *ERBB2*, and *AKT1*, against which specific inhibitors have been developed and explored in clinical trials, with the first PI3K inhibitor, alpelisib, recently approved in combination with fulvestrant for ER+/HER2-, *PIK3CA* mutant metastatic breast cancer (mBC) [2–4]. Activating mutations in *ESR1* acquired in response to aromatase inhibitor (AI) treatment have also emerged as an important genomic marker in breast cancer, with the ability to predict response to subsequent endocrine treatments [5, 6]. There is also growing development of more potent selective estrogen receptor inhibitors that can potentially overcome endocrine resistance associated with activating *ESR1* mutations [7]. Crucial to the success of these novel targeted approaches in breast cancer management is the integration of effective genomic testing programs into routine clinical practice.

Tumour biopsies remain the standard source to identify breast cancer–specific genomic alterations; however, improved methods are needed, especially in the metastatic setting. As an invasive procedure, biopsies inherently cause significant patient discomfort, may be inaccessible, or may yield limited amounts of tumour-derived DNA for testing. Importantly, both the spatial and temporal heterogeneity of a patient’s tumour cannot be captured through a single biopsy, as it can evolve during the course of treatment [8, 9]. This is particularly pertinent to mBC, in which it is not uncommon for genotyping to be performed on tumour-derived DNA collected several months or years prior to the onset of metastatic disease (e.g., from the time of the primary breast cancer diagnosis). Recently, circulating tumour DNA (ctDNA) testing has emerged as a minimally invasive approach to genomically profile a patient’s tumour from a simple blood draw [10, 11]. The ability to capture genomic heterogeneity and assess genomic changes in real time overcomes some key limitations of tissue biopsies. ctDNA analysis has been shown to recapitulate the genomic features of the underlying tumour and can profile multifocal clonal evolution in mBC over time [8, 9, 12].

The potential transformative clinical applications of ctDNA testing in cancer management include molecular profiling for treatment selection and disease monitoring. Several ctDNA-based companion diagnostic tests have now received United States Food and Drug Administration (FDA) approval, including for the detection of *PIK3CA* mutations in breast cancer. However, few prospective studies have been performed to date, and none in the mBC setting, which have integrated routine comprehensive ctDNA testing into clinical management [13–15]. The primary objective of this study was to establish an integrated prospective plasma DNA testing program for mBC patients to assess the feasibility and utility of routine ctDNA analysis to direct clinical management, comparing both droplet digital PCR (ddPCR) and next-generation sequencing (NGS) technologies. Here, we report our experience with the first 234 patients who underwent prospective testing on the plasma-based protocol, including the frequency of actionable alterations detected, a comparison of different ctDNA detection methods, the prognostic information gained from ctDNA testing, and the use of the ctDNA results to guide clinical management, including enrolment into clinical trials.

## Methods

### Study design and patient cohort

The Metastatic Breast Circulating Biomarker (MBCB) study was established at the Peter MacCallum Cancer Centre (Melbourne, Australia) in June 2015 to assess the feasibility and utility of applying routine comprehensive ctDNA profiling in mBC patients to guide clinical management. ctDNA results were provided to clinicians in real time, and the impact of these results on influencing clinical decisions was assessed by (1) the proportion of patients found to have actionable mutations using different ctDNA methodologies, (2) the proportion of patients for whom clinical management was changed based on the ctDNA results, and (3) the proportion of patients enrolled into clinical trials as a result of ctDNA testing. The primary objective of the study did not necessitate a prospective statistical analysis plan. Here, we detail our clinical experience over the first 3 years following the establishment of our testing program.

**Fig 1:**
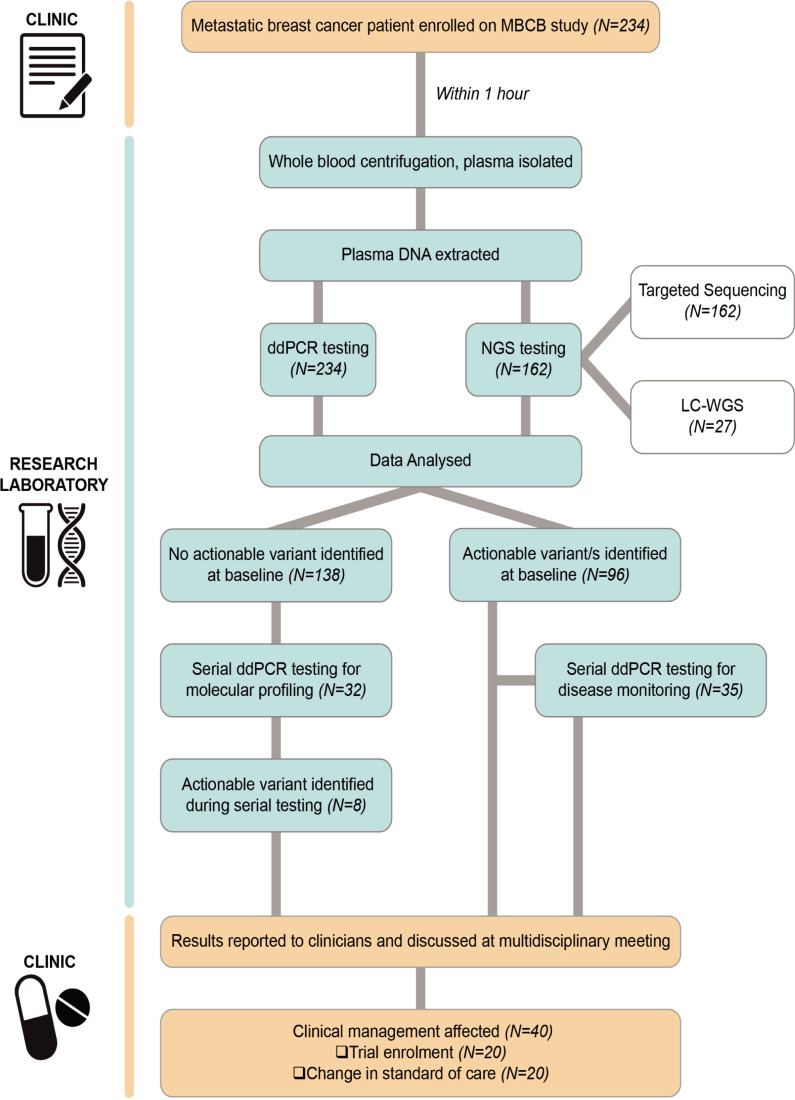
MBCB study workflow diagram. Workflow of ctDNA testing in the MBCB study. ctDNA, circulating tumour DNA; ddPCR, droplet digital PCR; LC-WGS, low-coverage whole-genome sequencing; MBCB, Metastatic Breast Circulating Biomarker; NGS, next-generation sequencing.

Eligible patients were consecutive women and men with newly diagnosed or previously established mBC of any histological subtype undergoing treatment. Institutional ethics approval was obtained (Peter MacCallum Cancer Centre Human Research Ethics Committee, project number 15/72) and patients provided written informed consent. At the discretion of treating clinicians, blood samples (30 ml each) were taken at study enrolment in all participants and then serially at intervals of 4 or more weeks for longitudinal testing, if deemed to be clinically relevant. Both ddPCR and NGS-based technologies were applied and compared for the prospective analysis of ctDNA. Results of ctDNA testing were discussed at multidisciplinary team meetings, where joint decisions were made regarding patient management, including accessibility to available clinical trials based on the genomic findings (Fig 1).

### Mutational analysis of ctDNA

Cell-free DNA was extracted from 2 ml of plasma with ddPCR analysis performed using a multiplex assay targeting 20 hotspot somatic mutations in *PIK3CA*, *ESR1*, *AKT1*, and *ERBB2* on the Bio-Rad QX-200 system. In parallel, targeted sequencing using a custom-developed panel of 394 amplicons across 39 genes frequently mutated in breast cancer (see S1 and S2 Tables for the list of genes on the panel and primer sequences), and low-coverage whole-genome sequencing (LC-WGS) for the analysis of copy number alterations (CNAs) was carried out as previously described [16, 17]. For a detailed description of the techniques and analysis, see S1 Text. A comparison of ddPCR and targeted sequencing was performed and reported as per the STARD 2015 reporting guideline for diagnostic accuracy studies (S1 Checklist).

### Statistical analysis

Associations between ctDNA levels and overall survival (OS) were tested using Cox regression models implemented with the *rms* R package. ctDNA levels were determined by the variant allele fraction (VAF) (for samples that underwent ddPCR and/or targeted panel sequencing) or a genome-wide tumour purity estimate (for samples that underwent LC-WGS). ctDNA levels were included in the Cox models with the use of natural cubic splines to allow for nonlinear association. We compared models with and without the use of natural cubic splines for ctDNA levels using the likelihood ratio test. There was a nonlinear association between the ctDNA levels and OS (*p* < 0.05) for LC-WGS and targeted panel sequencing; hence, we chose the model with spline terms. In contrast, the association for ddPCR was linear hence we did not include spline terms for this model. Optimal number of knots (degree of smoothing) were selected using the Bayesian information criterion. Concordance of VAFs between ddPCR and targeted sequencing was performed using a Spearman’s correlation.

## Results

### Cohort and study characteristics

**Fig 2:**
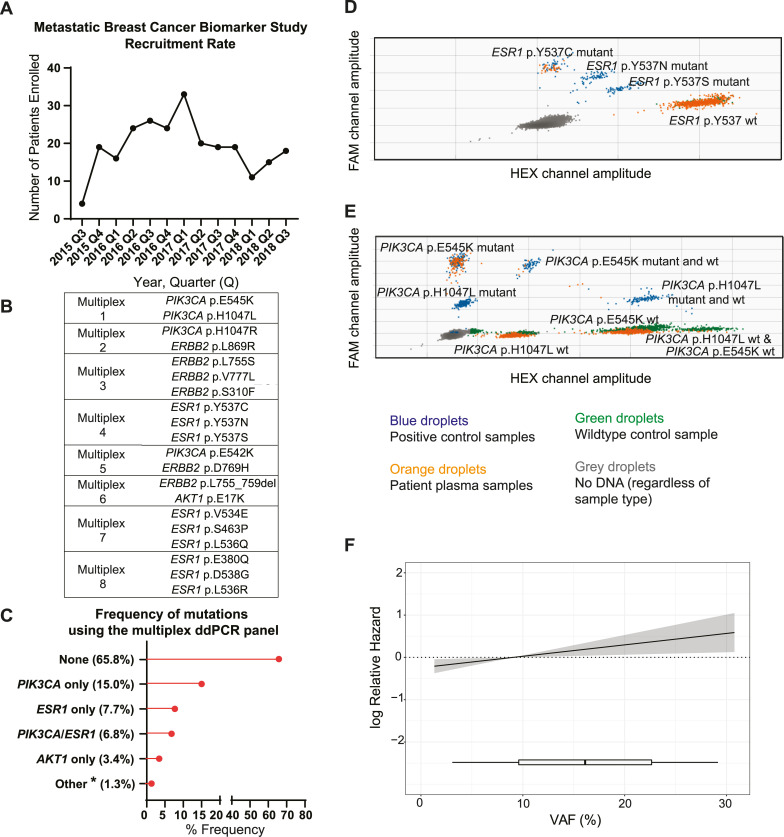
Patient recruitment and multiplex ddPCR testing of plasma samples at baseline. (A) Recruitment rate of patients on the MBCB study. (B) Layout of the multiplex ddPCR panel with 20 mutation-specific probes combined over 8 different reactions. (C) Frequency of mutations identified at baseline using the multiplex ddPCR panel in the 234 patients of the MBCB cohort. *One patient had both an *ESR1* and *AKT1* mutation detected; 1 patient had both a *PIK3CA* and *ERBB2* mutation detected; and 1 patient had an *ERBB2* mutation detected only. (D-E) Examples of the detection of specific mutations via the multiplex ddPCR assay using QuantaSoft software. (D) Results of multiplex reaction 4 show detection of the *ESR1* p.Y537C mutation in patient MBCB134, and (E) results of multiplex reaction 1 show detection of the *PIK3CA* p.E545K mutation in patient MBCB155. Orange droplets correspond to patient plasma samples (detected by either mutation [FAM]/wt probe [HEX] as indicated), blue droplets correspond to positive control samples, green droplets correspond to wt control samples (matched germline DNA), and grey droplets correspond to droplets with no DNA (regardless of sample type). (F) Linear Cox regression model showing the association between ctDNA VAF (as assessed by ddPCR) and overall survival (95% CI represented by shading). ctDNA, circulating tumour DNA; ddPCR, droplet digital PCR; MBCB, Metastatic Breast Circulating Biomarker; VAF, variant allele fraction; wt, wild type.

From June 2015 to October 2018 a total of 234 patients with mBC were enrolled on the MBCB study. All participants had a blood sample taken at enrolment (baseline sample), with a subset of the patients (*n* = 67, 28.6%) undergoing serial testing based on clinician choice (Fig 1). The cohort accurately reflected the full spectrum and distribution of breast cancer subtypes with 78% ER+/HER2-, 12% HER2+, and 10% with triple negative disease (Table 1) [18]. The median age of patients at the time of enrolment was 56 years, with the majority of participants having newly diagnosed mBC (*n* = 80; 34.2%) or disease progression following ≤2 prior lines of therapy (*n* = 89; 38%). The cohort exhibited variable sites of metastatic disease, including bone (*n* = 160; 68.4%), liver (*n* = 75; 32.1%), lung (*n* = 51; 21.8%), and central nervous system (CNS) (*n* = 9; 3.8%) disease. The median follow-up of study participants was 15 months (range 1–46). The rate of enrolment on the study initially increased steadily before plateauing to an average of 6 new patients per month (Fig 2A), reflecting the average number of new mBC patients seen at our centre.

**Table 1:** Patient characteristics of the MBCB cohort.

		Overall	ER+/HER2-	ER+/HER2+	ER-/HER2+	TNBC
*N* (% of overall)		234 (100%)	182 (77.8%)	14 (6.0%)	14 (6.0%)	24 (10.3%)
Gender, *N* (%)	Women	232 (99.1%)	180 (98.9%)	14 (100%)	14 (100%)	24 (100%)
	Men	2 (0.9%)	2 (1.1%)	0	0	0
Median age at diagnosis of mBC (years)		54 (28–80)	54 (30–80)	55 (39–73)	50 (28–73)	49 (33–74)
Disease sites at time of mBC diagnosis						
	Bone	160 (68.4%)	137 (75.3%)	10 (71.4%)	4 (28.6%)	9 (37.5%)
	Liver	75 (32.1%)	56 (30.8%)	4 (28.6%)	10 (71.4%)	5 (20.8%)
	Lung	51 (21.8%)	36 (19.3%)	4 (28.6%)	4 (28.6%)	7 (29.2%)
	CNS	9 (3.8%)	3 (1.6%)	4 (28.6%)	1 (7.1%)	1 (4.2%)
	Other	84 (35.9%)	63 (34.6%)	6 (42.9%)	2 (14.3%)	13 (54.2%)
Lines of prior treatment for mBC						
	0	80 (34.2%)	56 (30.8%)	5 (35.7%)	8 (57.1%)	11 (45.8%)
	1 or 2	89 (38.0%)	70 (38.5%)	5 (35.7%)	3 (21.4%)	11 (45.8%)
	3 or 4	35 (15.0%)	31 (17.0%)	2 (14.3%)	1 (7.1%)	1 (4.2%)
	≥5	30 (12.8%)	25 (13.7%)	2 (14.3%)	2 (14.3%)	1 (4.2%)
Median age at time of enrolment for ctDNA testing (years)		56 (28–83)	56 (30–83)	56 (41–76)	50 (28–81)	52 (34–76)
Duration of metastatic disease prior to ctDNA testing						
	<1year	102 (43.6%)	72 (39.6%)	6 (42.9%)	10 (71.4%)	14 (58.3%)
	1–5 years	107 (45.7%)	87 (47.8%)	7 (50.0%)	3 (21.4%)	10 (41.7%)
	6–13 years	25 (10.7%)	23 (12.6%)	1 (7.1%)	1 (7.1%)	0

Abbreviations: CNS, central nervous system; ctDNA, circulating tumour DNA; mBC, metastatic breast cancer; ER, estrogen receptor; HER2, human epidermal growth factor receptor 2; TNBC, triple negative breast cancer.

### Multiplexed ddPCR testing

We initially developed and optimized a multiplexed ddPCR assay for 20 targetable hotspot mutations in 4 selected genes (*PIK3CA*, *ESR1*, *AKT1*, and *ERBB2)* because of their direct relevance in mBC (Fig 2B). All plasma samples (baseline and serial, *n* = 432) were tested using the customised multiplexed ddPCR approach. Using ddPCR, 80/234 (34.2%) patients had ≥1 (ranging from 1 to 3) actionable mutation identified, with 52/234 (22.2%) patients having an alteration in *PIK3CA*, 35/234 (15.0%) in *ESR1*, 9/234 (3.8%) in *AKT1*, and 2/234 (0.9%) in *ERBB2 (*Fig 2C). Examples of the multiplex ddPCR analysis are shown in Fig 2D and 2E for patients with an *ESR1* p.Y537C and *PIK3CA* p.E545K mutation, respectively. We next examined if the ctDNA VAF could provide prognostic information. Indeed, increasing ctDNA levels based on VAF, were associated with inferior OS (Fig 2F).

### NGS

In addition to ddPCR testing, NGS testing was also applied on a subset of samples, using targeted panel sequencing (covering 39 genes recurrently mutated in breast cancer) and LC-WGS. Results were used to identify other putative mutations or CNAs that could direct clinical management and to assess the extent to which these methods could provide greater diagnostic performance compared to the multiplexed ddPCR assay. Targeted panel sequencing was conducted at baseline on the first 162 patients enrolled, with 159 (98.2%) passing sequencing analysis thresholds (S1 Text). LC-WGS was also carried out on samples from 27 patients (all of ER+/HER2- subtype).

#### Concordance between targeted panel sequencing and ddPCR

Across the 20 mutations analysed via ddPCR, targeted panel sequencing showed excellent concordance among the 159 patient samples tested at baseline. Out of the 73 mutations identified with either method, 58 (79.5%) were concordant between the two approaches. No false positives were detected by targeted sequencing. The major reason for the discordant cases were mutations detected by ddPCR but undetectable via targeted sequencing because they were below the analytical sensitivity of the assay, i.e., <1% VAF (S3 Table). A comparison of VAFs between the two methodologies across the 58 concordant mutations revealed a high degree of correlation (R^2^ = 0.89, S1 Fig). Targeted sequencing was also useful in interpreting the results in one instance where ddPCR produced an inconclusive result (S2A Fig). Through targeted sequencing, the patient was subsequently confirmed to have both a *PIK3CA* p.H1047L and *PIK3CA* p.H1047R mutation (S2B Fig).

#### Actionability of identified mutations from targeted panel sequencing

**Fig 3:**
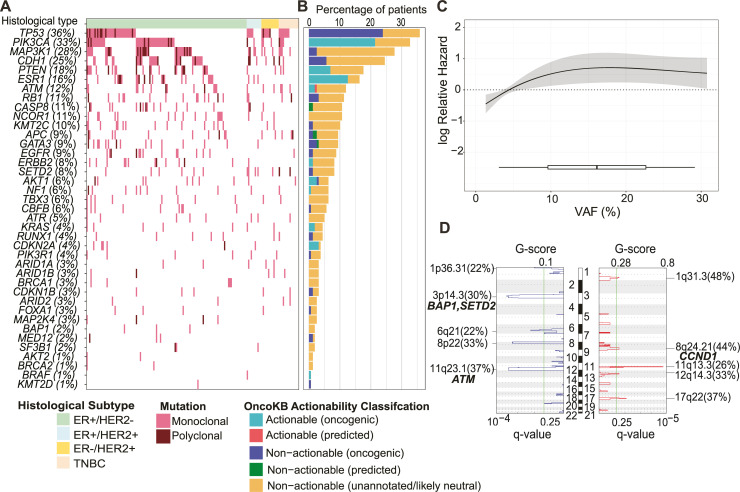
Next-generation sequencing of plasma samples at baseline. (A) Heatmap of mutations detected across all samples tested with the 39-gene targeted sequencing panel. Patients are ranked based on their histological subtype and the frequency of observed mutations. (B) Percentage of patients with actionable mutations detected from targeted sequencing based on the OncoKB classification database. (C) Cubic spline regression model showing the association between ctDNA VAF (as assessed by targeted sequencing) and overall survival (95% CI represented by shading). (D) GISTIC analysis showing copy number alterations from LC-WGS analysis (*n* = 27) with deleted (left) and amplified (right) chromosomal regions and genes indicated. Green line indicates the significance threshold for copy number alterations (q-value 0.25). ctDNA, circulating tumour DNA; ER, estrogen receptor; HER2, human epidermal growth factor receptor 2; LC-WGS, low-coverage whole-genome sequencing; TNBC, triple negative breast cancer; VAF, variant allele fraction.

At least 1 mutation was identified in 147/159 (92.5%) patients (Fig 3A, S4 Table), with an average of 4 mutations/sample (range 0–18) by targeted sequencing. At least 1 mutation was detected in all 39 genes represented on the panel (range 1–73) with the most frequently altered genes being *TP53* (36.0% of patients), *PIK3CA* (32.7%), *MAP3K1* (27.7%), *CDH1* (24.5%), *PTEN* (17.6%), and *ESR1* (16.4%), closely resembling that of other studies that have undertaken genomic profiling of breast cancer [19–22]. A recent study by Bertucci and colleagues compared genomic profiles of metastatic and early breast cancer, revealing a number of driver genes to be more frequently mutated in the metastatic setting [23]. We conducted a similar analysis, comparing our ctDNA findings in the mBC setting to that reported in the early breast cancer setting from The Cancer Genome Atlas (TCGA). As expected, our findings confirm overall higher mutational burden in the metastatic setting, with several key driver genes being significantly more mutated as compared to early stage breast cancer (*ESR1*, *KMT2C*, *MAP3K1*, *PTEN*, *CASP8*, *EGFR*, *AKT1*, *ATM*, and *RB1*) (S3 Fig).

We next assessed the potential influence of the identified mutations on clinical practice using the OncoKB database, revealing 96/627 (15.3%) of identified mutations as actionable (including oncogenic and likely oncogenic) [24]. Because of the increased breadth of gene coverage on the targeted sequencing panel, 62/159 (39.0%) patients were identified to have an actionable mutation (Fig 3B, S4 Table), representing a modest improvement over the proportion of patients (34.2%) with an identifiable actionable mutation through the focused multiplex ddPCR approach alone. This was primarily driven by the identification of additional mutations in *PTEN*, *ATM*, *NF1*, *CDKN2A*, *KRAS*, and *BRAF*. The average mutational VAF per patient across the cohort ranged from 1.1% to 65.9%, with a median of 4.1%. Consistent with our previous findings with ddPCR (Fig 2F), we found that an increasing ctDNA VAF by targeted sequencing was associated with inferior OS (Fig 3C).

#### CNAs revealed from ctDNA LC-WGS

We next assessed whether additional information could be gained from ctDNA analysis via LC-WGS, given that our targeted sequencing approach did not allow for the detection of CNAs. The LC-WGS analysis was performed in ER+/HER2- cases only, and it identified a number of recurrent copy number–altered regions (Fig 3D). In particular, chromosome regions 11q13.3 (including the *CCND1* gene, amplified in 26% of the cohort) and 17q22 were frequently amplified across the cohort. Interestingly, an *ERBB2* amplification was also identified in one case through the LC-WGS ctDNA analysis, which had not been identified from analysis of multiple previous tissue biopsies (discussed further below). We also found several chromosomal regions to be frequently deleted in our cohort, including 11q23 (*ATM*, 33%) and 3p14.3 (*BAP1* and *SETD2*, 26%), which are known important tumour suppressor genes. ctDNA levels based on LC-WGS were also a significant predictor of outcome, with increasing tumour purity associated with inferior OS (S4 Fig).

### Impact of ctDNA testing on clinical management and trial enrolment

As guidelines for the clinical interpretation of ctDNA-based results in mBC have not yet been established, all results from baseline and/or serial ctDNA testing were provided to clinicians, and joint decisions regarding patient management were made at a dedicated weekly mBC multidisciplinary team meeting following discussion of the molecular results (Fig 1). Across all ctDNA testing methods in the 234 patients at baseline, at least 1 actionable alteration was identified in 96 patients (41.0%, Fig 1). Subsequent serial testing in patients between 6 months and 2 years after an initial negative result at baseline detected the emergence of an actionable mutation in 8 more patients, resulting in a total of 104/234 (44.4%) patients in whom an actionable mutation was found.

**Fig 4:**
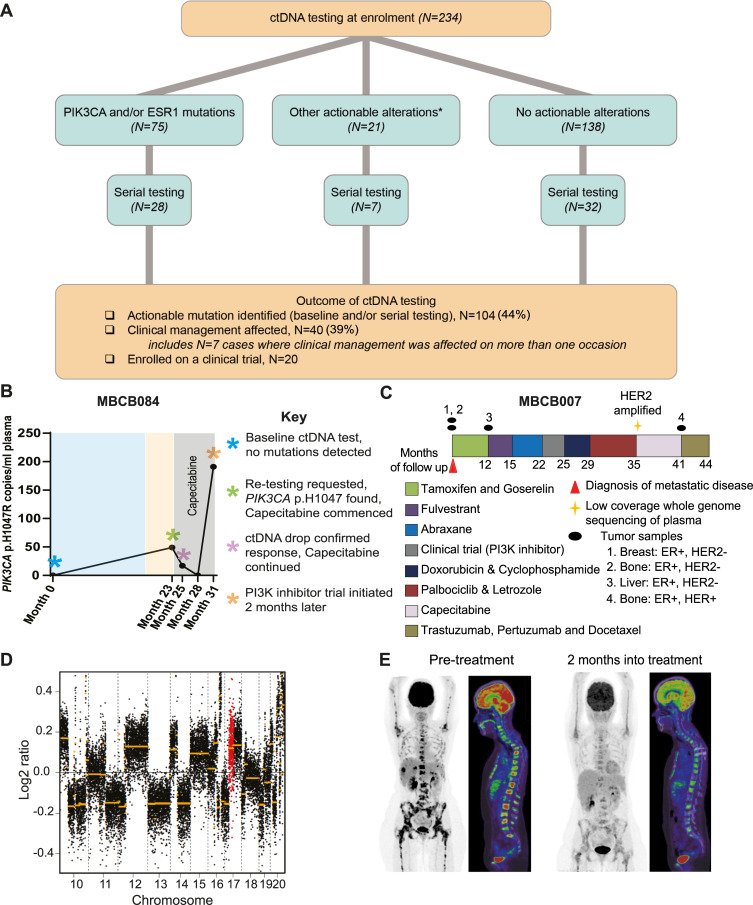
ctDNA testing affects clinical management in mBC patients. (A) Summary of all clinical decisions affected across the MBCB cohort by ctDNA testing using ddPCR and/or NGS methods (both at baseline and through serial testing). *Other actionable mutations were found in genes *AKT1*, *ERBB2*, *PTEN*, *ATM*, *NF1*, *CDKN2A*, *KRAS*, and *BRAF*. (B) Serial ctDNA testing results from patient MBCB084. No actionable mutations were detected at baseline (month 0). Approximately 2 years later, at initiation of first-line chemotherapy (capecitabine), a *PIK3CA* p. H1047R mutation was detected using the multiplex ddPCR assay. Treatment was continued after 2 months based on a drop in *PIK3CA* p. H1047R ctDNA levels (despite a mixed response on imaging). At the time of disease progression on capecitabine, the patient was enrolled on a PI3K inhibitor trial as a result of the *PIK3CA* p. H1047R mutation detected in plasma. (C) Treatment timeline for patient MBCB007 following diagnosis of mBC, with the duration of each treatment, time points of plasma sequencing, and sequential ER/HER2 classification from tumour biopsy samples. (D) Copy number profile of plasma taken from patient MBCB007 post treatment with palbociclib and letrozole revealed an amplification of the chromosome 17 region containing *ERBB2* (highlighted in red). (E) CT and PET images of MBCB007, before and after subsequent treatment with HER2-targeted therapy (trastuzumab, pertuzumab, and docetaxel), showing near complete metabolic response 2 months after starting treatment. CT, computed tomography; ctDNA, circulating tumour DNA; ddPCR, droplet digital PCR; ER, estrogen receptor; HER2, human epidermal growth factor receptor 2; mBC, metastatic breast cancer; MBCB, Metastatic Breast Circulating Biomarker; NGS, next-generation sequencing; PET, positron emission tomography.

The ctDNA result directly affected clinical management in 40 patients (39% of those in whom an actionable mutation had been identified and 17% of the entire cohort), including on multiple occasions in 7 cases in which serial testing was undertaken (Fig 4A). This was defined by enrolment on an available clinical trial or a change in standard therapy as a direct consequence of the ctDNA result (S5 Table). A total of 20 patients with a *PIK3CA* mutation were enrolled on a clinical trial of a PI3K inhibitor following the ctDNA result (NCT02506556 or NCT02437318) [25]. *ESR1* mutations typically informed choice of endocrine treatment, with AI treatment avoided in 23 patients and fulvestrant avoided in 1 patient based on presence of the *ESR1* p.Y537S mutation [7]. A total of 64 patients, who had an actionable mutation detected at baseline or serially, did not have their clinical management affected by ctDNA testing (S6 Table). The three most common reasons why clinical management was unaffected were no availability of the relevant targeted therapy or appropriate clinical trial at the time of ctDNA testing (32.8%), loss to follow-up (31.3%), or patients not meeting clinical trial eligibility criteria (25.0%).

#### Serial testing of plasma in clinical management

Blood samples were collected and tested serially for 67/234 patients (28.6%, S5 Fig), at the request of the treating clinician. Serial testing occurred as frequently as once per month, and patients had between 2 and 12 time points tested. Of the 96 patients with a mutation detected at baseline, clinicians ordered serial testing for 35 of these cases for the purpose of treatment monitoring, often to compare the ctDNA results with the findings obtained from imaging (e.g., in the setting of equivocal radiological findings). In these cases, the vast majority of serial testing was performed via a singleplex ddPCR assay, testing for the specific mutations previously detected at baseline. In parallel, clinicians also ordered serial testing for 32 patients who had no mutations detected via ddPCR at baseline, for identification of the emergence of actionable mutations during their disease course. In all cases, the serial testing was performed using the multiplex ddPCR assay, and as detailed above, retesting led to a positive result in 8 cases (25%).

To provide an insight into how clinical management was altered by the use of ctDNA testing, we have described 2 cases. In the first case, MBCB084, retesting was performed at initiation of treatment with capecitabine, almost 2 years after the baseline result gave no actionable mutation (Fig 4B). The activating *PIK3CA* p.H1047R mutation was detected at the serial time point and then monitored during the time course of treatment. Two months after treatment on capecitabine, standard imaging showed a mixed response; however, a fall in *PIK3CA* p.H1047R ctDNA levels resulted in continuation of treatment. The patient continued to respond to capecitabine but had progression after 7 months, after which they were enrolled on a PI3K inhibitor clinical trial based on the *PIK3CA* mutation detected via ctDNA.

In the second case, MBCB007 (Fig 4C), who was initially diagnosed with ER+/HER2- metastatic disease via routine tumour biopsy immunohistochemistry (IHC) and in situ hybridization (S6A Fig and S6B Fig), we detected emergence of a subclone harbouring *ERBB2* amplification through LC-WGS of ctDNA following treatment with a CDK4/6 inhibitor in combination with letrozole (Fig 4D). To confirm this finding, a bone biopsy was performed on which IHC and silver in situ hybridization (SISH) analysis was performed, which indeed confirmed the HER2 amplification (S6C and S6D Fig). Consequently, HER2-targeted treatment was initiated, resulting in near complete metabolic response after 2 months (Fig 4E).

## Discussion

In this study, we established a comprehensive ctDNA-based genomic profiling program to guide treatment decisions in mBC. This included the establishment of a workflow for sample collection; the development of rapid, robust, and accurate testing methods; and the delivery of results to clinicians to guide patient management. Identification of actionable alterations was possible in 44% of patients tested, and patient management was affected in 39% of these individuals. Through our program, we also performed serial analysis of plasma for the purposes of disease monitoring or to identify the emergence of actionable mutations. In several cases, de novo actionable mutations were identified at times of relapse on treatment, highlighting the importance of serial ctDNA testing for effective patient management. Finally, in addition to comprehensive molecular profiling, the quantitative information provided from ctDNA analysis also provided important prognostic information. High ctDNA levels, as detected by any methodology, were associated with inferior survival in mBC, and this finding has now been demonstrated consistently across several studies [26, 27].

Our improved understanding of the genomic landscape of metastatic disease, accompanied by increasing availability of molecularly targeted therapies, is setting a new paradigm in the therapeutic options for mBC patients [2–4]. These developments highlight that reliable and comprehensive real-time genomic testing will be essential to guide patient management and match targeted therapy selection. Prospective studies that demonstrate the utility of ctDNA approaches in different cancer settings are needed to drive routine use of ctDNA forward in the clinic. To the best of our knowledge, our study is the first to do this in the context of mBC, showing the feasibility, value, and importance of this approach to complement routine patient care. Several recent studies have established ctDNA testing programs to direct clinical management and clinical trial enrolment, but these have all been pan-cancer studies and included only limited breast cancer cases [13–15]. Here, we provide a bespoke breast cancer–specific ctDNA approach that provides a flexible template that could be widely adopted for incorporation of ctDNA testing into clinical practice.

Currently, there is no ‘standard’ methodology for ctDNA analysis, and few prior studies have performed cross-platform comparisons. A key strength of our study is that we were able to evaluate a range of different ctDNA-based detection methods. We chose to examine 4 breast cancer gene targets using a multiplex ddPCR assay because of the availability of matched targeted therapies and the rapid turnaround time of testing. For the application of NGS-based targeted sequencing, we developed an in-house breast cancer–specific panel, which led to an increase in the frequency of mutations detected compared to the use of a pan-cancer panel [14, 28]. It resulted in a modest improvement in the number of actionable alterations identified when compared to our ddPCR assay, owing to the broader genomic coverage, but this had limited additional impact on clinical management because of the lack of corresponding therapeutics routinely available. In parallel, our identification of potentially actionable CNAs from LC-WGS also highlights the importance of incorporating CNA detection into future testing workflows. Although the choice of technology will, in part, depend on the technical expertise available at a testing site, factors such as the cost of an assay, number of samples for testing, and the accessibility of targeted therapies may influence the choice of an assay and how this is implemented diagnostically at specific sites.

The findings from our prospective testing program revealed a number of advantages over current practice. To date, the use of tumour biopsies has been the primary source to identify mutations from mBC patients. Previous studies have already shown good concordance to detect mutations using plasma compared to matching tumour biopsies [29]. However, the genomic analysis of DNA from tumour samples is known to have challenges associated with biopsy retrieval and histological review, as well as the potential for longer turnaround times. In various cancer centres, turnaround time is often reported in weeks, which is in excess of desirable practice [30]. In contrast, our results highlight that ctDNA analysis can bypass these tissue testing challenges, as we were able to deliver results in a turnaround time as short as 2 days when necessary. Importantly, the testing of tumour-derived DNA, usually from formalin-fixed sections, can have a high rate of failure because of insufficient tumour content, DNA input/quality, or technical failures. ctDNA analysis, on the other hand, had a much higher rate of successful testing (i.e., 98% from our targeted sequencing), negating the need for repeat testing that can extend turnaround times.

The main limitations of the study were that it was conducted at a single site and 31.3% of participants were lost to follow-up. Importantly, the impact of ctDNA testing on survival outcomes was not assessed. Future studies will be required to document both the clinical and health economic impact of routine ctDNA testing in the mBC setting.

Overall, our study shows that comprehensive genomic profiling using breast cancer–specific ctDNA testing is a feasible approach that can reveal specific genomic changes underlying metastatic disease. Our experience supports routine implementation of ctDNA testing to complement tumour testing in mBC, by enabling actionable targets to be captured in real time to direct clinical management.

## Supporting information

S1 Checklist STARD Checklist. (PDF)

S1 Text Supporting methods. (DOCX)

S1 Fig Concordance of variant allele fractions between ddPCR and targeted panel sequencing. All mutations detected by both methods are included, with each dot representing a single mutation. Statistical analysis was performed using a Spearman’s correlation. Linear regression line: y = 1.00x + 0.13. ddPCR, droplet digital PCR.
(PDF)

S2 Fig Targeted amplicon sequencing detects co-occurring *PIK3CA* p.H1047R and p.H1047L mutations in patient MBCB165. (A) Singleplex ddPCR assay for the *PIK3CA* p.H1047L mutation does not unequivocally detect this mutation, because although there is positive scatter detected for this mutation, there is no overlap between positive control (SUM159 cell line DNA, blue) and test sample droplets (MBCB165 plasma DNA, orange). Green droplets; wild-type *PIK3CA* at the p.H1047 locus detected in SUM159 cells; grey droplets: contain no PCR product (regardless of sample type). (B) Screenshot using the Integrative Genomic Viewer of targeted sequencing results for MBCB165, with reads from nucleotide 178952085 on chromosome 3 highlighted, showing presence of an alteration from base A>T (*PIK3CA* p.H1047R) and A>G (*PIK3CA* p.H1047L) on both replicates (subpanels a and b correspond to each replicate). As a result of this finding and confirmation of multiple hotspot *PIK3CA* mutations, the patient was enrolled on a PI3K inhibitor clinical trial. ddPCR, droplet digital PCR; MBCB, Metastatic Breast Circulating Biomarker.
(PDF)

S3 Fig Comparison of gene mutational frequencies between the metastatic breast cancers from the MBCB cohort and early breast cancers from the TCGA cohort. Scatterplots show mutational frequencies (percentage of patients) between the two cohorts, with each dot representing 1 of the 39 genes from the targeted sequencing panel used in the MBCB cohort. Genes with significantly different mutational frequencies between the two cohorts are labelled (adjusted *P* value < 0.001). Statistics are based on a two-sided Fisher’s exact test, corrected for multiple testing. MBCB, Metastatic Breast Circulating Biomarker; TCGA, The Cancer Genome Atlas.
(PDF)

S4 Fig Relationship between overall survival and tumour purity using LC-WGS of patient plasma. LC-WGS, low-coverage whole-genome sequencing.
(PDF)

S5 Fig Summary of serial ctDNA testing undertaken in the study. ctDNA, circulating tumour DNA.
(PDF)

S6 Fig Histological analysis of tumour samples from patient MBCB007 validated the *ERBB2* gene amplification detected from LC-WGS of ctDNA. Tumour samples 1, 3, and 4 refer to the tumour samples referenced in Fig 4C. IHC analysis on breast tissue at the time of metastatic disease diagnosis and of a liver biopsy at time of development of liver metastases did not show any HER2 overexpression (A and B, respectively). IHC and SISH analyses on bone tissue following progression on palbociclib and letrozole revealed positive staining for HER2 (C and D, respectively). HER2, human epidermal growth factor receptor 2; IHC, immunohistochemistry; SISH, silver in situ hybridization.
(PDF)

S7 Fig Limit of detection for the multiplex ddPCR assay. Shown is multiplex well 1 as a representative example. (A) Both *PIK3CA* p.E545K and *PIK3CA* p.H1047L probes in multiplex well 1 can detect these mutations in MCF7 and SUM159 cell lines, respectively. (B-F) A titration series of varying VAFs ranging from 5% VAF to 0.1% VAF using a Horizon Discovery Control (Multiplex I cfDNA Reference Standard Set-HD780) reference standard containing the *PIK3CA* p.E545K mutation. 5 ng was used as an input with this mutation detected down to 0.1% VAF. No false positive droplets were detected with (G) WT cfDNA reference (Horizon Discovery Control [HD780, Part No.:HD776]) or with (H) input containing no DNA (nuclease-free water). Scatterplots show fluorescent detection of individual droplets with FAM channel (blue) corresponding to mutant DNA (*PIK3CA* p.E545K or *PIK3CA* p.H1047L, as indicated) and HEX channel (green) corresponding to WT DNA. Orange represents double droplets containing both mutant and WT DNA. Grey represents droplets that did not contain PCR product. Each plot is an overlay of 2 replicates. ddPCR, droplet digital PCR; VAF, variant allele fraction; WT, wild type.
(PDF)

S1 Table Genes represented on the targeted sequencing panel. (DOCX)

S2 Table List of primers of the targeted sequencing panel. (XLSX)

S3 Table List of mutations detected via ddPCR versus targeted panel sequencing in the *PIK3CA*, *AKT1*, *ERBB2*, and *ESR1* hotspots screened. ddPCR, droplet digital PCR.
(XLSX)

S4 Table Variants called from targeted panel sequencing. (XLSX)

S5 Table Summary of how ctDNA testing impacted clinical management. ctDNA, circulating tumour DNA.
(XLSX)

S6 Table Summary of why clinical management was unaffected in patients with actionable mutations detected. (XLSX)

S7 Table NextSeq sequencing metrics (LC-WGS). LC-WGS, low-coverage whole-genome sequencing.
(XLSX)

## Abbreviations

AI: aromatase inhibitor
CNA: copy number alteration
CNS: central nervous system
CT: computed tomography
ctDNA: circulating tumour DNA
ddPCR: droplet digital PCR
ER: estrogen receptor
FDA: United States Food and Drug Administration
HER2: human epidermal growth factor receptor 2
IHC: immunohistochemistry
LC-WGS: low-coverage whole-genome sequencing
mBC: metastatic breast cancer
MBCB: Metastatic Breast Circulating Biomarker
NGS: next-generation sequencing
OS: overall survival
PET: positron emission tomography
SISH: silver in situ hybridization
TCGA: The Cancer Genome Atlas
TNBC: triple negative breast cancer
VAF: variant allele fraction
wt: wild type
