# Supplementary material for: Circulating tumour DNA in metastatic breast cancer to guide clinical trial enrolment and precision oncology: A cohort study
Source: PLoS Med. 2020 Oct 1;17(10):e1003363. doi: 10.1371/journal.pmed.1003363 (PMC7529214; doi:10.1371/journal.pmed.1003363)
Supplement: S1 Checklist — (PDF) [file pmed.1003363.s001.pdf]

Section & Topic	No	Item	Reported on page #
**TITLE OR ABSTRACT**			
	**1**	Identification as a study of diagnostic accuracy using at least one measure of accuracy (such as sensitivity, specificity, predictive values, or AUC)	Abstract, paragraph 2
**ABSTRACT**			
	**2**	Structured summary of study design, methods, results, and conclusions (for specific guidance, see STARD for Abstracts)	Abstract, paragraphs 1 to 4
**INTRODUCTION**			
	**3**	Scientific and clinical background, including the intended use and clinical role of the index test	Introduction, paragraphs 1 to 3
	**4**	Study objectives and hypotheses	Introduction, paragraph 3
**METHODS**			
Study design	**5**	Whether data collection was planned before the index test and reference standard were performed (prospective study) or after (retrospective study)	Methods, paragraph 2
Participants	**6**	Eligibility criteria	Methods, paragraph 2
	**7**	On what basis potentially eligible participants were identified (such as symptoms, results from previous tests, inclusion in registry)	Methods, paragraph 2
	**8**	Where and when potentially eligible participants were identified (setting, location and dates)	Methods, paragraphs 1 and 2
	**9**	Whether participants formed a consecutive, random or convenience series	Methods, paragraph 2
Test methods	**10a**	Index test, in sufficient detail to allow replication	Methods, paragraph 3
	**10b**	Reference standard, in sufficient detail to allow replication	Supporting methods
	**11**	Rationale for choosing the reference standard (if alternatives exist)	Supporting methods
	**12a**	Definition of and rationale for test positivity cut-offs or result categories of the index test, distinguishing pre-specified from exploratory	Supporting methods
	**12b**	Definition of and rationale for test positivity cut-offs or result categories of the reference standard, distinguishing pre-specified from exploratory	Supporting methods
	**13a**	Whether clinical information and reference standard results were available to the performers/readers of the index test	Supporting methods
	**13b**	Whether clinical information and index test results were available to the assessors of the reference standard	Supporting methods
Analysis	**14**	Methods for estimating or comparing measures of diagnostic accuracy	Methods, paragraph 1, supporting methods
	**15**	How indeterminate index test or reference standard results were handled	Supporting methods, and S2 figure
	**16**	How missing data on the index test and reference standard were handled	Results, paragraphs 3 and 4
	**17**	Any analyses of variability in diagnostic accuracy, distinguishing pre-specified from exploratory	Results, paragraph 4
	**18**	Intended sample size and how it was determined	Methods, paragraph 1
**RESULTS**			
Participants	**19**	Flow of participants, using a diagram	Figure 1
	**20**	Baseline demographic and clinical characteristics of participants	Results, paragraph 1 and Table 1
	**21a**	Distribution of severity of disease in those with the target condition	Results, paragraph 1 and Table 1
	**21b**	Distribution of alternative diagnoses in those without the target condition	N/A
	**22**	Time interval and any clinical interventions between index test and reference standard	N/A

Test results	**23**	Cross tabulation of the index test results (or their distribution) by the results of the reference standard	S3 Table
	**24**	Estimates of diagnostic accuracy and their precision (such as 95% confidence intervals)	Results, paragraphs 2 and 6
	**25**	Any adverse events from performing the index test or the reference standard	N/A
**DISCUSSION**			
	**26**	Study limitations, including sources of potential bias, statistical uncertainty, and generalisability	Discussion, paragraph 5
	**27**	Implications for practice, including the intended use and clinical role of the index test	Discussion, paragraphs 2, 3 and 4
**OTHER INFORMATION**			
	**28**	Registration number and name of registry	Methods, paragraph 2
	**29**	Where the full study protocol can be accessed	N/A
	**30**	Sources of funding and other support; role of funders	Funding
