# Supplementary material for: Circulating tumour DNA in metastatic breast cancer to guide clinical trial enrolment and precision oncology: A cohort study
Source: PLoS Med. 2020 Oct 1;17(10):e1003363. doi: 10.1371/journal.pmed.1003363 (PMC7529214; doi:10.1371/journal.pmed.1003363)
Supplement: S1 Text — (DOCX) [file pmed.1003363.s002.docx]

**S1 TEXT. SUPPORTING METHODS**

**Blood collection and plasma DNA extraction**

Blood samples were collected in EDTA tubes and processed within 1 hour after collection. Processing involved initial centrifugation at 1,600 g for 10 min to separate plasma from peripheral blood cells followed by a further centrifugation step at 20,000 g for 10 min to pellet any remaining cells and/or debris. The plasma was then stored at -80° C until DNA extraction. DNA was extracted from up to 2 ml of plasma using the QIAmp Circulating Nucleic Acid Kit (Qiagen) according to manufacturer’s instructions. The DNA was eluted into 50 µl buffer AVE (Qiagen) and stored at -20◦ C. Matched normal germline DNA (from MNL or saliva) was obtained from study participants at enrolment to enable confirmation of somatic status of identified genetic events. In the case of saliva, ∼2 ml were collected in an Oragene Saliva Collection Kit (DNA Genotek, OG500). DNA was then extracted from saliva using the prepIT L2P kit (DNA Genotek, PTL2P45), according to the manufacturer’s protocols. DNA was extracted from the peripheral blood mononuclear layer (MNL) cells of the buffy coat using the DNA Blood and Tissue kit (Qiagen, 69504), using the manufacturer's protocols. Clinical information from patients was available to the laboratory researchers who performed subsequent plasma testing.

**Multiplex ddPCR of ctDNA**

ddPCR analysis was performed using the Bio-Rad Droplet Digital PCR system following manufacturer’s protocols. Allele-specific quantitative PCR assays to specifically detect 20 hotspot somatic mutations and corresponding wild-type alleles in *PIK3CA, ESR1, AKT1* and *ERBB2* were either custom designed or commercially obtained. The primary purpose of testing for mutations in *PIK3CA* was for potential enrolment of patients onto one of several PI3K-alpha inhibitor clinical trials open at our centre. Testing for mutations in *AKT1* and *ERBB2* was aimed at potentially obtaining compassionate access for eligible patients to an AKT1 or pan-HER inhibitor (neratinib), respectively, or enrolment on any trials with these agents open at our centre. Finally, mutations in *ESR1* were tested to guide choice of endocrine treatments in ER+ patients.

To achieve higher throughput, the panel was multiplexed across an 8-well assay with either 2 or 3 different probes combined in the same reaction (Fig 2B). This multiplexed panel was used to screen for the presence/absence of mutations. Any detected mutations were then validated and quantified (as VAF or absolute copies/ml plasma) using singleplex assays for those specific mutations, as previously described [1]. Multiplex reactions were run in duplicate with a 5 µl input per well, while singleplex reactions were run in triplicate with a 5-10uL input per well. Positive and negative controls for each of the mutations were run in parallel for each run. Positive controls used were either cell line controls or synthetic DNA fragments (gBlocks®, Integrated DNA Technologies) containing the specific mutation. Negative controls consisted of both MNL-derived DNA samples known to be wildtype for a particular mutation and nuclease free water included in each run. For both multiplex and singleplex reactions, ctDNA was defined as detectable if there was ≥ 1 copy of mutant DNA detected in each of two duplicate reactions**.** Data analysis was carried out using the QuantaSoft Software, version 1.7 (Bio-Rad). For multiplex reactions, a test sample was considered positive for a particular mutation (and consequently verified via singleplex assay) if the overlap between QuantaSoft images for the test sample and positive control showed detected droplets in the same cluster for the mutant DNA (Fig 2D and E). For the multiplexed panel, a limit of detection (LOD, defined as ≥1 positive droplet in both replicates tested) was determined at 0.1% VAF given a DNA input amount of ~5ng (S7 Figure).

**Targeted panel sequencing of circulating tumor DNA**

Targeted panel sequencing was performed on plasma-derived DNA and matched germline DNA using the 48.48 Access Array™ system (Fluidigm). As previously described, a panel of 394 amplicons with average amplicon length of 164 bases was designed across 39 genes (S1 and S2 Table*)* [2]. The panel was designed to cover genes recurrently mutated in mBC (including actionable targets) with the aim of identifying a mutation in as many patients as possible. For the purpose of selecting candidate genes, we interrogated the literature for key genomic profiling studies of breast cancer published at the time of panel development [3-10]. The findings across these studies were combined and both the reported mutational frequencies in each study and the number of studies each gene appeared in were considered to arrive at the final list of 39 candidate genes. Using the COSMIC database, regions of these genes containing mutations previously reported in breast cancer were selected and amplicons were designed to cover these regions. Primers for the 394 amplicons were multiplexed (4 to 6 amplicon reactions per well), thus limiting the experimental workflow to two 48-well plates of the Fluidigm Access Array. Multiplexed amplicons were matched as closely as possible for GC content and melting temperature, while ensuring their genomic locations are >1000 nucleotides apart (preferably located on different genes), thus ensuring performance efficiency across the panel. Both plasma and germline DNA samples (derived from either mononuclear cell layer (MNL) or saliva) were sequenced for each patient, with all samples run in duplicate to control for reproducibility in variants called. The libraries were then sequenced with the NextSeq or MiSeq system (Illumina) using paired end sequencing with v2 150-bp kits. The mean targeted sequencing coverage was 818-fold for plasma and 1220-fold for germline samples. Bcl2fastq V.2.17.1 was used to perform sample demultiplexing and to convert BCL files generated from the NextSeq/MiSeq instrument into FastQ files containing short-read data. Using the primer sequences that are present in the data, short reads were first assigned to their respective amplicon. Samples were annotated through the ‘PathOS’ pipeline: a web-based variant management system [11]. To restrict our analysis to high confidence variants and control for sequencing and/or PCR artefacts, samples were removed from analysis if at least 1 replicate had a read coverage depth <100 in either germline or plasma samples, resulting in 3 patient samples being removed from the analysis. Furthermore, variants were only called if they were absent in both germline replicates but appeared in both technical replicates at >1% VAF (unless previously detected via ddPCR, in which case there was no restriction applied on VAF cutoff). To further eliminate artefacts, variants not previously reported in the COSMIC database were removed from the final list if they appeared in >1.5% of sequenced samples (defined as panel variance). Variants with a high global allele frequency (>1.0%) in the 1000 genomes database were also removed.

**Low coverage whole genome sequencing of circulating tumor DNA**

Standard LC-WGS was performed on selected patient plasma samples as previously described [1]. Libraries were prepared and sequenced on the Illumina NextSeq500 platform (paired-end 75 bp) according to standard protocol. Mean depth of coverage was 6X (detailed sequencing metrics have been provided in S7 Table). Copy number analysis was performed using ichorCNA (v0.1.0) with bin size 1000kb, mapping quality threshold 20 and other parameters set as default. Segmented data from ichorCNA was input into GISTIC (v2.0.23) with an amplification/deletion threshold > 0.1, confidence level 0.90, Q-value threshold 0.25 and other parameters set as default. For each significant lesion called by GISTIC, the fraction of patients with the alteration was calculated from the *all_lesions.conf_90.txt* output. Genes with recurrent aberrations were identified from the wide peaks called by GISTIC. For each gene, the fraction of patients with the alteration was calculated from *all_threshold.by_genes.txt*.

**Comparison of mutational frequencies between MBCB and TCGA cohorts**

To determine which genes are more/less frequently mutated between the MBCB cohort (using targeted sequencing data) and The Cancer Genome Atlas (TCGA) primary breast cancer cohort, we performed a Fisher’s exact test. Previously curated somatic variants for all TCGA breast cancer patients (977 patients) were downloaded from the TCGA archive (https://portal. gdc.cancer.gov/legacy-archive/files/50d6fb1d-5bb1-4a30-9e91-6d45bd9b1c3f ) and associated clinical data was downloaded from the TCGA data portal. All patients classified as stage four/x or not reported were removed from further analysis, with 941 patients remaining. Multiple hypothesis testing was adjusted for using the Benjamini-Hochberg method.

**Data Availability**

The sequencing data that support the findings of this study has been deposited into the sequence read archive, which is hosted by the National Centre for Biotechnology Information. The BioProject accession number is PRJNA578569.

**References to supporting methods**

1. Yeh P, Hunter T, Sinha D, Ftouni S, Wallach E, Jiang D, et al. Circulating tumour DNA reflects treatment response and clonal evolution in chronic lymphocytic leukaemia. Nat Commun. 2017;8:14756.

2. Lok SW, Whittle JR, Vaillant F, Teh CE, Lo LL, Policheni AN, et al. A phase 1b dose-escalation and expansion study of the BCL-2 inhibitor venetoclax combined with tamoxifen in ER and BCL-2-positive metastatic breast cancer. 2019:CD-18-1151.

3. Banerji S, Cibulskis K, Rangel-Escareno C, Brown KK, Carter SL, Frederick AM, et al. Sequence analysis of mutations and translocations across breast cancer subtypes. Nature. 2012;486(7403):405-9.

4. Craig DW, O'Shaughnessy JA, Kiefer JA, Aldrich J, Sinari S, Moses TM, et al. Genome and transcriptome sequencing in prospective metastatic triple-negative breast cancer uncovers therapeutic vulnerabilities. Mol Cancer Ther. 2013;12(1):104-16.

5. Ellis MJ, Ding L, Shen D, Luo J, Suman VJ, Wallis JW, et al. Whole-genome analysis informs breast cancer response to aromatase inhibition. Nature. 2012;486(7403):353-60.

6. Kandoth C, McLellan MD, Vandin F, Ye K, Niu B, Lu C, et al. Mutational landscape and significance across 12 major cancer types. Nature. 2013;502(7471):333-9.

7. Lawrence MS, Stojanov P, Mermel CH, Robinson JT, Garraway LA, Golub TR, et al. Discovery and saturation analysis of cancer genes across 21 tumour types. Nature. 2014;505(7484):495-501.

8. Ng CK, Schultheis AM, Bidard FC, Weigelt B, Reis-Filho JS. Breast cancer genomics from microarrays to massively parallel sequencing: paradigms and new insights. J Natl Cancer Inst. 2015;107(5).

9. Shah SP, Roth A, Goya R, Oloumi A, Ha G, Zhao Y, et al. The clonal and mutational evolution spectrum of primary triple-negative breast cancers. Nature. 2012;486(7403):395-9.

10. Stephens PJ, Tarpey PS, Davies H, Van Loo P, Greenman C, Wedge DC, et al. The landscape of cancer genes and mutational processes in breast cancer. Nature. 2012;486(7403):400-4.

11. Doig KD, Fellowes A, Bell AH, Seleznev A, Ma D, Ellul J, et al. PathOS: a decision support system for reporting high throughput sequencing of cancers in clinical diagnostic laboratories. Genome Med. 2017;9(1):38.
