# Supplementary figures and images for: Circulating tumour DNA in metastatic breast cancer to guide clinical trial enrolment and precision oncology: A cohort study
Source: PLoS Med. 2020 Oct 1;17(10):e1003363. doi: 10.1371/journal.pmed.1003363 (PMC7529214; doi:10.1371/journal.pmed.1003363)

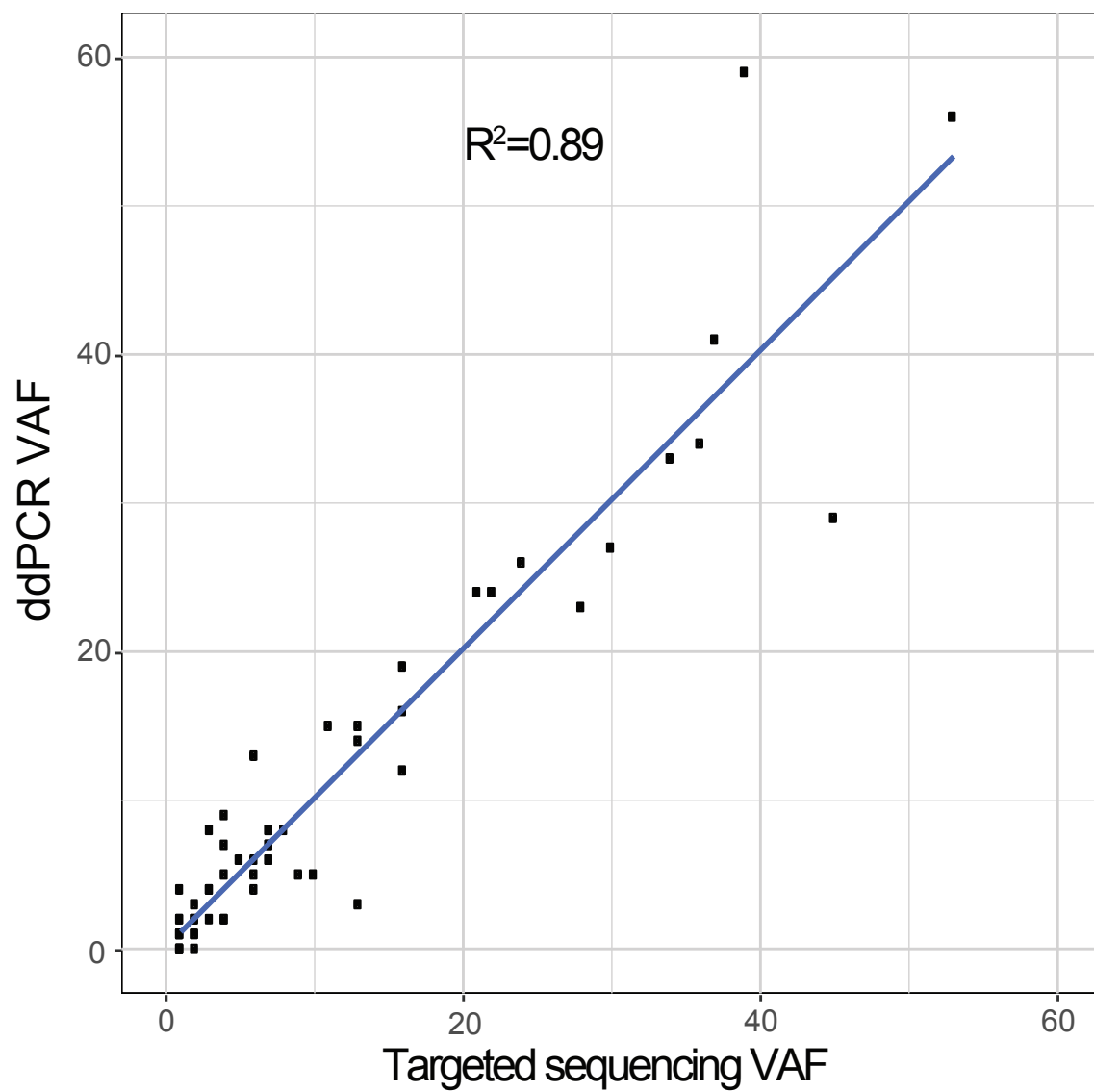

Supplement: S1 Fig — All mutations detected by both methods are included, with each dot representing a single mutation. Statistical analysis was performed using a Spearman’s correlation. Linear regression line: y = 1.00x + 0.13. ddPCR, droplet digital PCR. (PDF) [file pmed.1003363.s003.pdf]

A

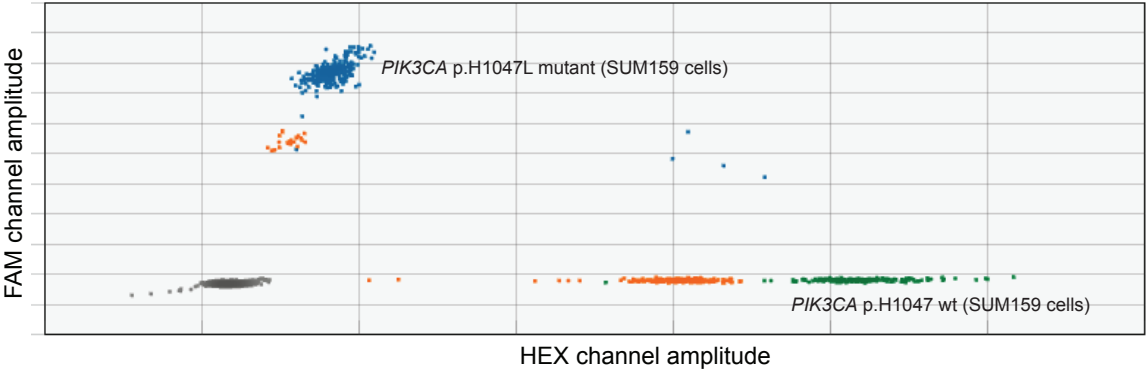

B

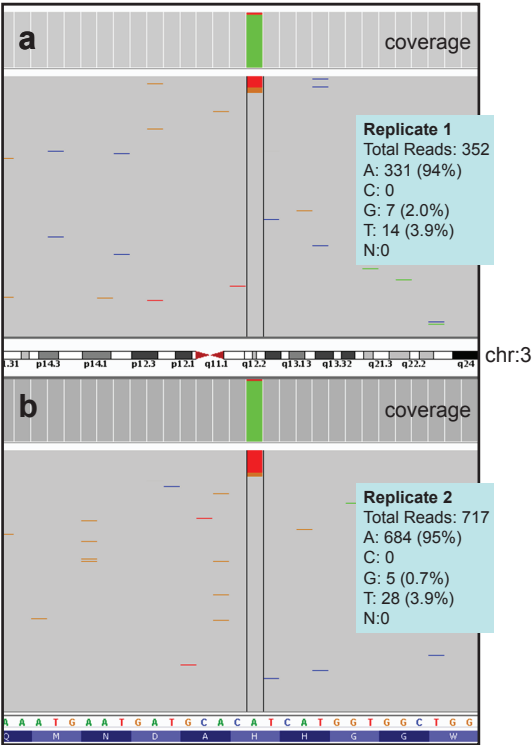

Supplement: S2 Fig — (A) Singleplex ddPCR assay for the PIK3CA p.H1047L mutation does not unequivocally detect this mutation, because although there is positive scatter detected for this mutation, there is no overlap between positive control (SUM159 cell line DNA, blue) and test sample droplets (MBCB165 plasma DNA, orange). Green droplets; wild-type PIK3CA at the p.H1047 locus detected in SUM159 cells; grey droplets: contain no PCR product (regardless of sample type). (B) Screenshot using the Integrative Genomic Viewer of targeted sequencing results for MBCB165, with reads from nucleotide 178952085 on chromosome 3 highlighted, showing presence of an alteration from base A>T (PIK3CA p.H1047R) and A>G (PIK3CA p.H1047L) on both replicates (subpanels a and b correspond to each replicate). As a result of this finding and confirmation of multiple hotspot PIK3CA mutations, the patient was enrolled on a PI3K inhibitor clinical trial. ddPCR, droplet digital PCR; MBCB, Metastatic Breast Circulating Biomarker. (PDF) [file pmed.1003363.s004.pdf]

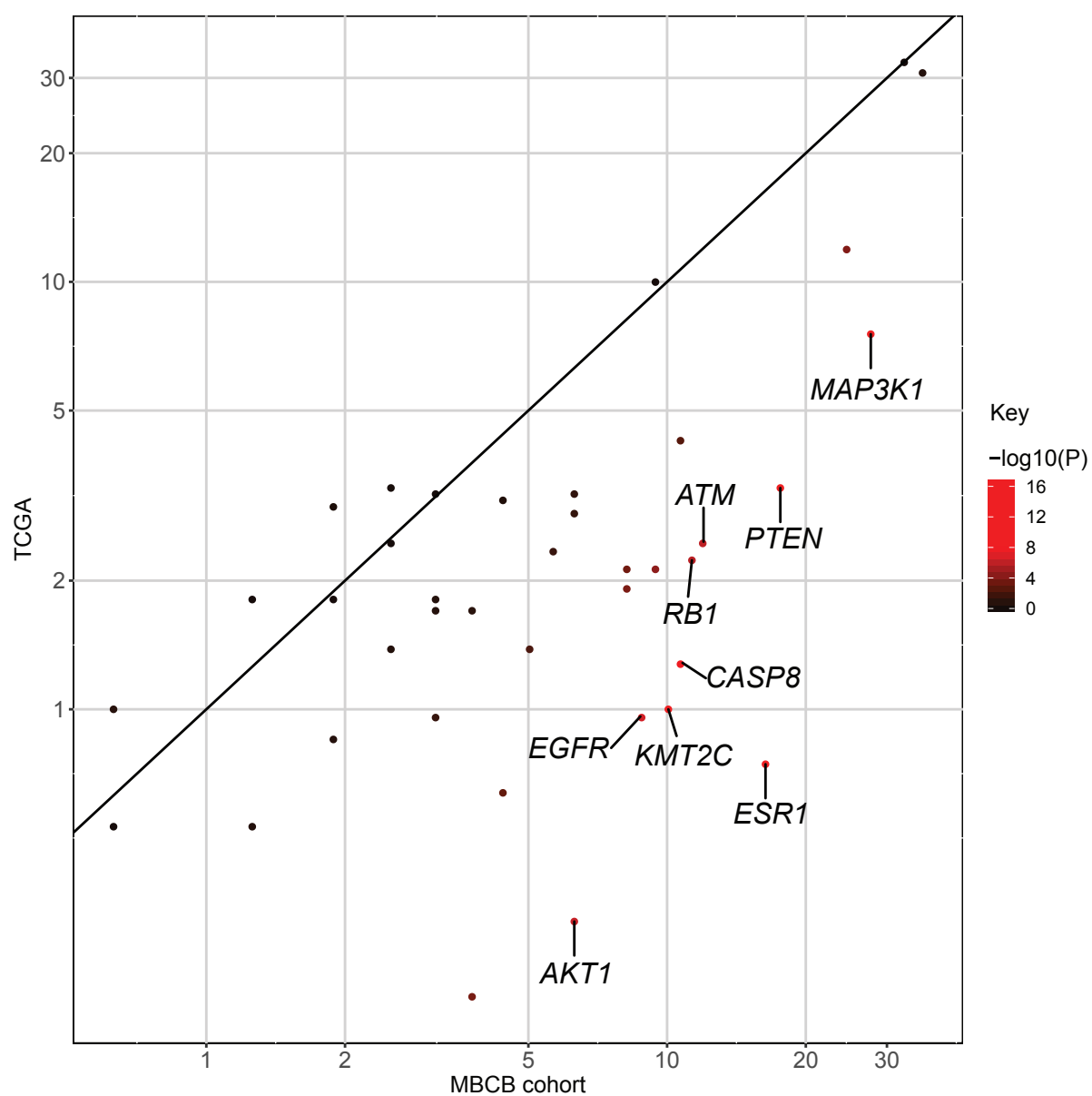

Supplement: S3 Fig — Scatterplots show mutational frequencies (percentage of patients) between the two cohorts, with each dot representing 1 of the 39 genes from the targeted sequencing panel used in the MBCB cohort. Genes with significantly different mutational frequencies between the two cohorts are labelled (adjusted P value < 0.001). Statistics are based on a two-sided Fisher’s exact test, corrected for multiple testing. MBCB, Metastatic Breast Circulating Biomarker; TCGA, The Cancer Genome Atlas. (PDF) [file pmed.1003363.s005.pdf]

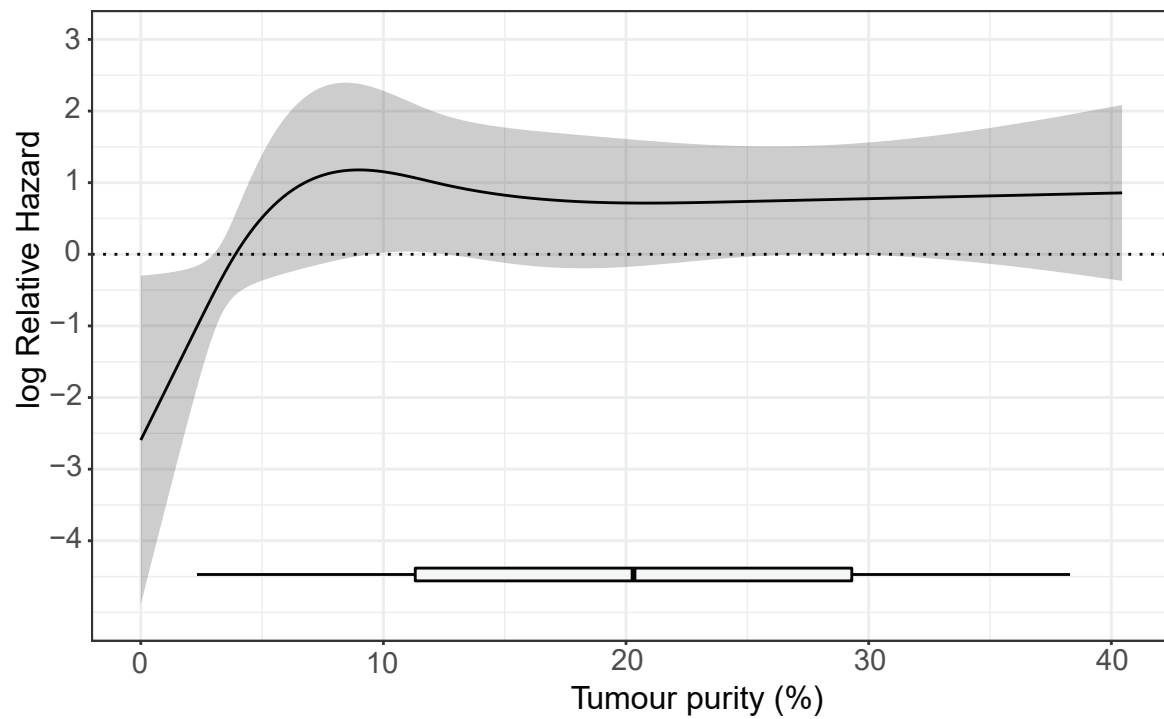

Supplement: S4 Fig — LC-WGS, low-coverage whole-genome sequencing. (PDF) [file pmed.1003363.s006.pdf]

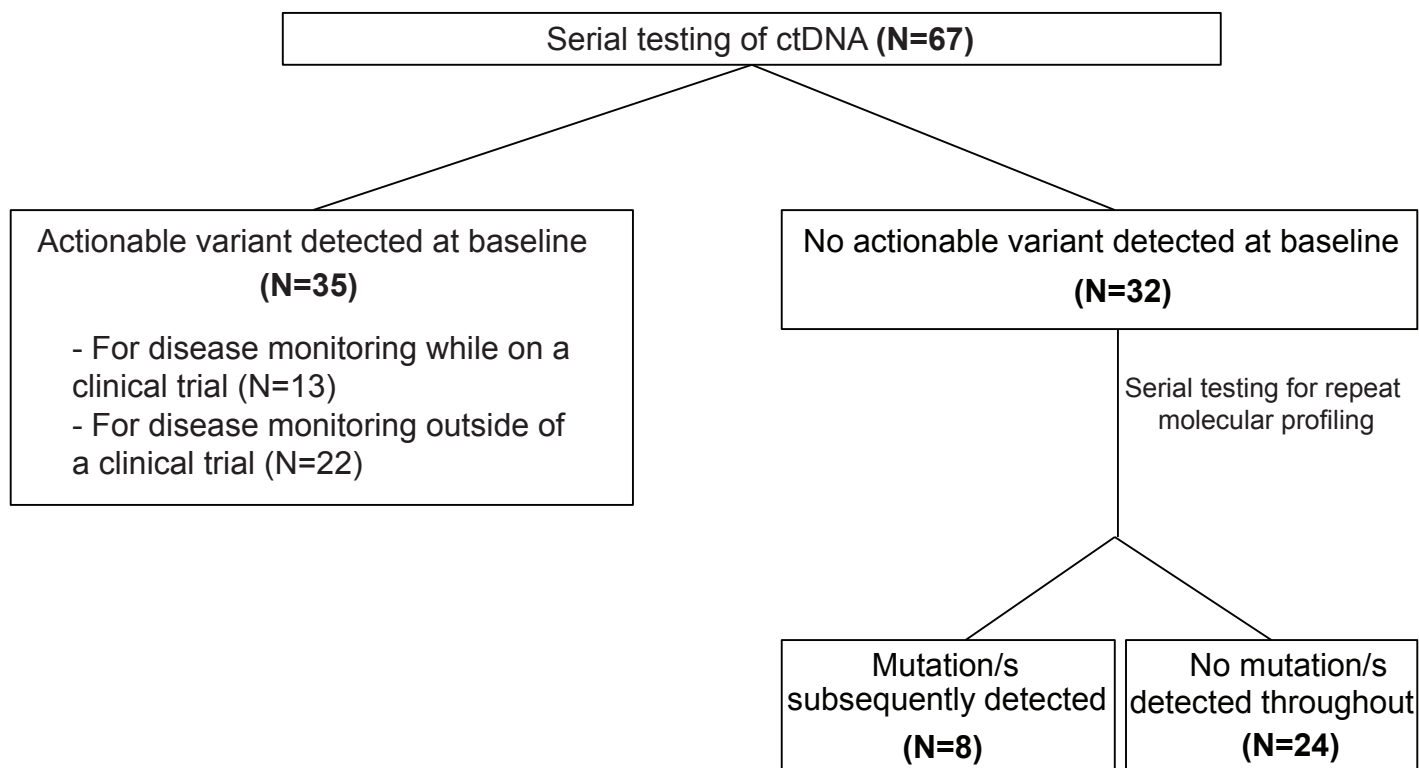

Supplement: S5 Fig — ctDNA, circulating tumour DNA. (PDF) [file pmed.1003363.s007.pdf]

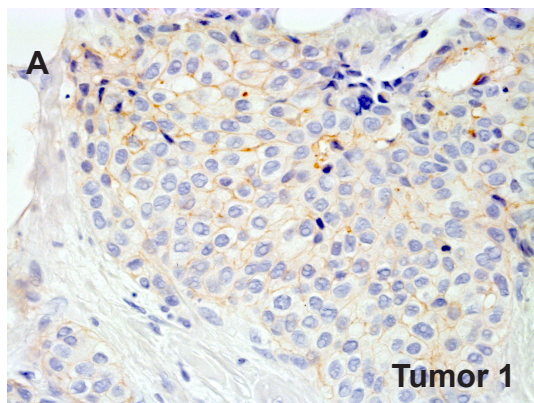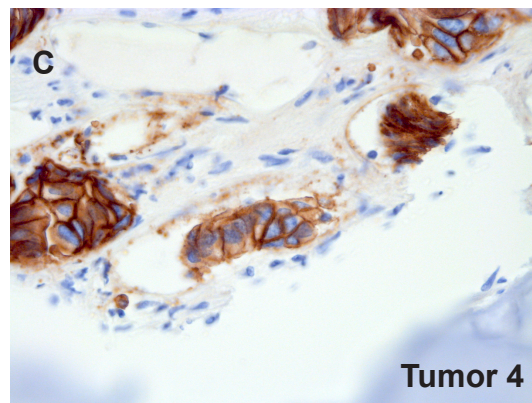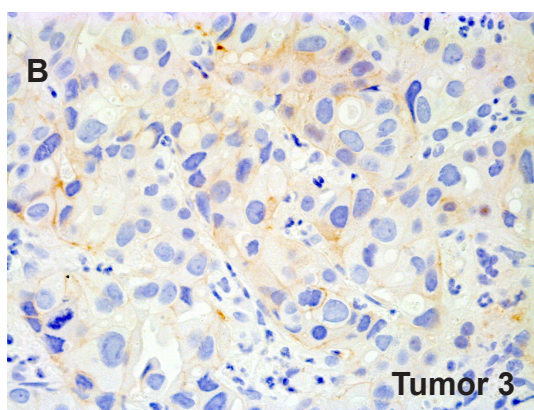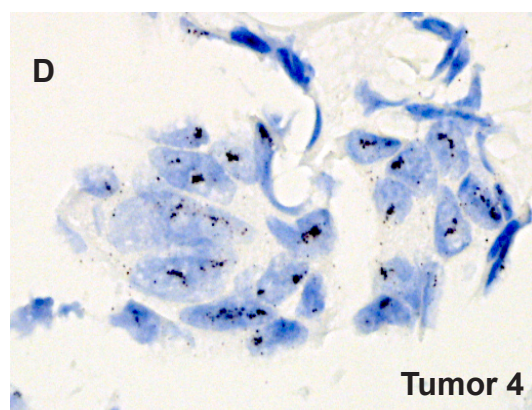

Supplement: S6 Fig — Tumour samples 1, 3, and 4 refer to the tumour samples referenced in Fig 4C. IHC analysis on breast tissue at the time of metastatic disease diagnosis and of a liver biopsy at time of development of liver metastases did not show any HER2 overexpression (A and B, respectively). IHC and SISH analyses on bone tissue following progression on palbociclib and letrozole revealed positive staining for HER2 (C and D, respectively). HER2, human epidermal growth factor receptor 2; IHC, immunohistochemistry; SISH, silver in situ hybridization. (PDF) [file pmed.1003363.s008.pdf]

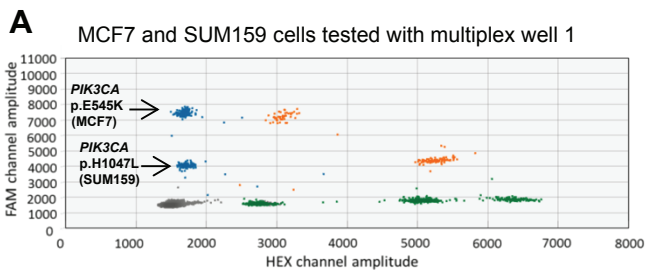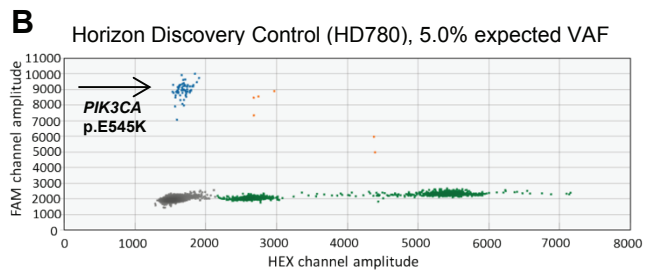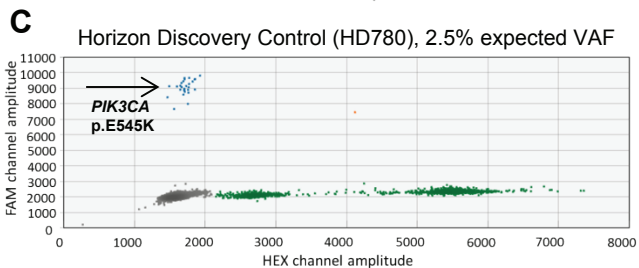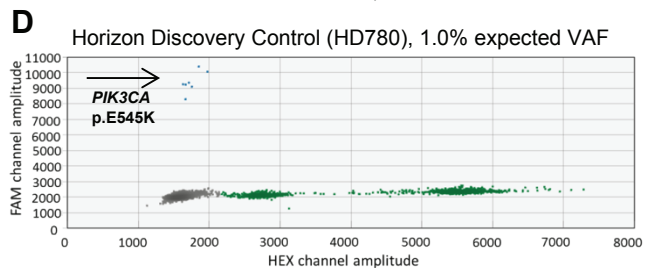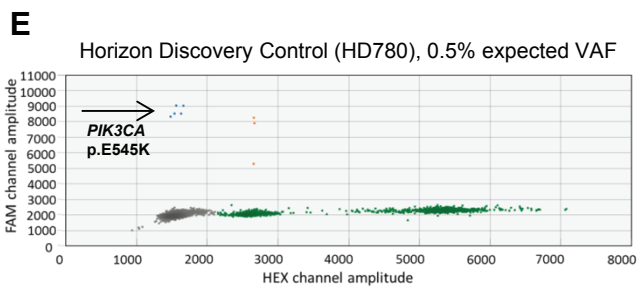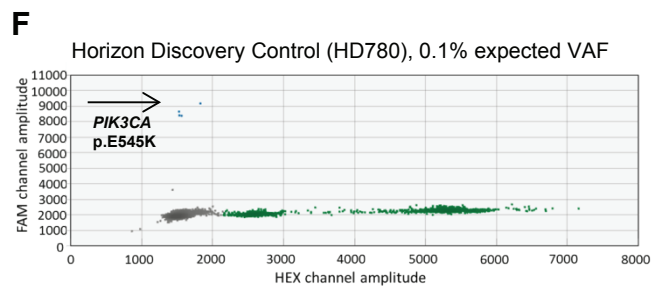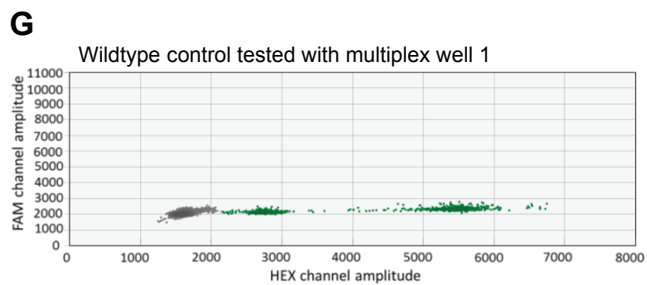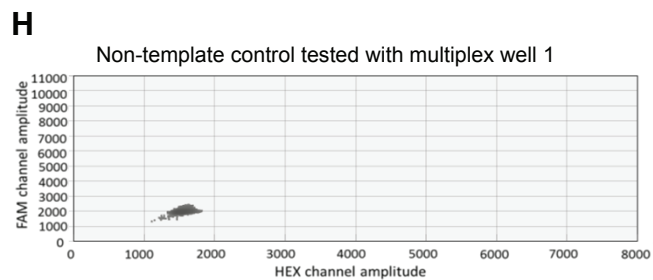

Supplement: S7 Fig — Shown is multiplex well 1 as a representative example. (A) Both PIK3CA p.E545K and PIK3CA p.H1047L probes in multiplex well 1 can detect these mutations in MCF7 and SUM159 cell lines, respectively. (B-F) A titration series of varying VAFs ranging from 5% VAF to 0.1% VAF using a Horizon Discovery Control (Multiplex I cfDNA Reference Standard Set-HD780) reference standard containing the PIK3CA p.E545K mutation. 5 ng was used as an input with this mutation detected down to 0.1% VAF. No false positive droplets were detected with (G) WT cfDNA reference (Horizon Discovery Control [HD780, Part No.:HD776]) or with (H) input containing no DNA (nuclease-free water). Scatterplots show fluorescent detection of individual droplets with FAM channel (blue) corresponding to mutant DNA (PIK3CA p.E545K or PIK3CA p.H1047L, as indicated) and HEX channel (green) corresponding to WT DNA. Orange represents double droplets containing both mutant and WT DNA. Grey represents droplets that did not contain PCR product. Each plot is an overlay of 2 replicates. ddPCR, droplet digital PCR; VAF, variant allele fraction; WT, wild type. (PDF) [file pmed.1003363.s009.pdf]
