# Supplementary material for: Circulating tumour DNA in metastatic breast cancer to guide clinical trial enrolment and precision oncology: A cohort study
Source: PLoS Med. 2020 Oct 1;17(10):e1003363. doi: 10.1371/journal.pmed.1003363 (PMC7529214; doi:10.1371/journal.pmed.1003363)
Supplement: S1 Table — (DOCX) [file pmed.1003363.s010.docx]

| **S1 Table. Genes represented on the targeted sequencing panel** | | | |
| --- | --- | --- | --- |
| **Gene** | **No. of Amplicons** | **Gene** | **No. of Amplicons** |
| *MAP3K1* | 43 | *NF1* | 9 |
| *CDH1* | 24 | *SETD2* | 9 |
| *PIK3CA* | 21 | *MED12* | 8 |
| *GATA3* | 18 | *RUNX1* | 7 |
| *TP53* | 17 | *ATR* | 7 |
| *NCOR1* | 17 | *ARID1B* | 7 |
| *MLL3 (KMT2C)* | 17 | *CBFB* | 6 |
| *PTEN* | 16 | *CDKN1B* | 5 |
| *EGFR* | 14 | *KMT2D* | 5 |
| *ARID1A* | 14 | *FOXA1* | 4 |
| *PIK3R1* | 11 | *CDKN2A* | 4 |
| *MAP2K4* | 11 | *ARID2* | 4 |
| *ATM* | 11 | *BAP1* | 4 |
| *CASP8* | 11 | *KRAS* | 3 |
| *RB1* | 10 | *SF3B1* | 2 |
| *BRCA2* | 10 | *AKT1* | 2 |
| *APC* | 10 | *TBX3* | 2 |
| *ESR1* | 10 | *AKT2* | 1 |
| *BRCA1* | 10 | *BRAF* | 1 |
| *ERBB2* | 9 |  |  |
